# Exploring the hidden treasure in arid regions: pseudocereals as sustainable, climate-resilient crops for food security

**DOI:** 10.3389/fpls.2025.1756967

**Published:** 2026-01-12

**Authors:** Ramya Manoharan, Sugandha Asthana, Chythra Somanathan Nair, Trupti Gokhale, Drishya Nishanth, Abdul Jaleel, Neeru Sood

**Affiliations:** 1 Department of Integrative Agriculture, College of Agriculture and Veterinary Medicine, United Arab Emirates University, Al Ain, United Arab Emirates; 2 Department of Biotechnology, Birla Institute of Technology and Science Pilani, Dubai Campus, Dubai International Academic City, Dubai, United Arab Emirates

**Keywords:** alternative crop, pseudocereals, nutritional value, food security, climate resilience, arid regions, sustainable agriculture, GWAS

- The reference for “Alencar and de Carvalho Oliveira, 2023” was erroneously written as “Alencar, N., and de Carvalho Oliveira, L. (2023). Advances in pseudocereals: Crop cultivation, food application, and consumer perception. *Bioactive Compounds Health Dis*. 6, 295–312. doi: 10.31989/bchd.v6i12.1223”. It should be “Alencar, N., and de Carvalho Oliveira, L. (2019). Advances in pseudocereals: Crop cultivation, food application, and consumer perception. *Bioactive Compounds Health Dis*. 6, 295–312. doi: 10.1007/978-3-319-78030-6_63”.
- The reference for “Alvarez-Jubete et al., 2010” was erroneously written as “Alvarez-Jubete, L., Arendt, E. K., and Gallagher, E. (2010). Nutritive value of pseudocereals and their increasing use as functional gluten-free ingredients. *Trends Food Sci Technol*. 21, 106–113. doi: 10.1016/j.tifs.2009.10”. It should be “Alvarez-Jubete, L., Arendt, E. K., and Gallagher, E. (2010). Nutritive value of pseudocereals and their increasing use as functional gluten-free ingredients. *Trends Food Sci Technol*. 21, 106–113. doi: 10.1016/j.tifs.2009.10.014”.
- The reference for “Brouns and Shewry, 2022” was erroneously written as “Brouns, F., and Shewry, P. R. (2022). Cereal grains and health: A review of the evidence. *Front. Nutr.* 9. doi: 10.3389/fnut.2022.908676”. It should be “Brouns, F., and Shewry, P. R. (2022). Do gluten peptides stimulate weight gain in humans? *Nutr. Bull.* 47 (2), 186-198. doi: 10.1111/nbu.12558”.
- The reference for “Curti etal., 2017” was erroneously written as “Curti, R. N., Sajama, J., and Ortega-Baes, P. (2017). Setting conservation priorities for Argentina’s pseudocereal crop wild relatives. *Biol. Conserv.* 209, 349–355. doi: 10.1016/j.biocon.2017.03.006”. It should be “Curti, R. N., Sajama, J., and Ortega-Baes, P. (2017). Setting conservation priorities for Argentina’s pseudocereal crop wild relatives. *Biol. Conserv.* 209, 349–355. doi: 10.1016/j.biocon.2017.03.008”.
- The reference for “de la Barca etal., 2010” was erroneously written as “de la Barca, A. M. C., Rojas-Martınez, M. E., Islas-Rubio, A. R., and Cabrera-Chavez, F. (2010). Gluten-free breads and cookies of raw and popped amaranth flours with attractive technological and nutritional qualities. *Plant Foods Hum. Nutr.* 65, 241–246. doi: 10.1007/s11130-010-0178-0”. It should be “de la Barca, A. M. C., Rojas-Martınez, M. E., Islas-Rubio, A. R., and Cabrera-Chavez,F. (2010). Gluten-free breads and cookies of raw and popped amaranth flours with attractive technological and nutritional qualities. *Plant Foods Hum. Nutr*. 65, 241–246. doi: 10.1007/s11130-010-0187-z”.
- The reference for “Derbali et al., 2021” was erroneously written as “Derbali, W., Manaa, A., Spengler, B., Goussi, R., Abideen, Z., Ghezellou, P., et al. (2021). Comparative proteomic approach to study the salinity effect on the growth of two contrasting quinoa genotypes. *Plant Physiol. Biochem.* 163, 215–229. doi: 10.1016/j.plaphy.2021.03.036”. It should be “Derbali, W., Manaa, A., Spengler, B., Goussi, R., Abideen, Z., Ghezellou, P., et al.(2021). Comparative proteomic approach to study the salinity effect on the growth of two contrasting quinoa genotypes. *Plant Physiol. Biochem.* 163, 215–229. doi: 10.1016/j.plaphy.2021.03.055”.
- The reference for “Devi and Chrungoo, 2017” was erroneously written as “Devi, R. J., and Chrungoo, N. K. (2017). Evolutionary divergence in Chenopodium and validation of SNPs in chloroplast rbcL and matK genes by allele-specific PCR for development of Chenopodium quinoa-specific markers. *Crop J.* 5, 32–42. doi: 10.1016/j.cj.2016.06.001”. It should be “Devi, R. J., and Chrungoo, N. K. (2017). Evolutionary divergence in Chenopodium and validation of SNPs in chloroplast rbcL and matK genes by allele-specific PCR for development of Chenopodium quinoa-specific markers. *Crop J.* 5, 32–42. doi: 10.1016/j.cj.2016.06.019”.
- The reference for “Fang et al., 2014” was erroneously written as “Fang, Z. W., Qi, R., Li, X. F., and Liu, Z. X. (2014). Ectopic expression of FaesAP3, a Fagopyrum esculentum (Polygonaceae) AP3 orthologous gene rescues stamen development in an Arabidopsis ap3 mutant. *Gene* 550, 200–206. doi: 10.1016/j.gene.2014.08.019”. It should be “Fang, Z. W., Qi, R., Li, X. F., and Liu, Z. X. (2014). Ectopic expression of FaesAP3, a *Fagopyrum esculentum* (Polygonaceae) AP3 orthologous gene rescues stamen development in an Arabidopsis ap3 mutant. *Gene* 550, 200–206. doi: 10.1016/j.gene.2014.08.029”.
- The reference for “Gorinstein et al., 2002” was erroneously written as “Gorinstein, S., Pawelzik, E., Delgado-Licon, E., Haruenkit, R., Weisz, M., and Trakhtenberg, S. (2002). Characterisation of pseudocereal and cereal proteins by protein and amino acid analyses. J. Sci Food Agric. 82, 886–891. doi: 10.1002/jsfa.1131”. It should be “Gorinstein, S., Pawelzik, E., Delgado-Licon, E., Haruenkit, R., Weisz, M., and Trakhtenberg, S. (2002). Characterisation of pseudocereal and cereal proteins by protein and amino acid analyses. *J. Sci Food Agric*. 82, 886–891. doi: 10.1002/jsfa.1120”.
- The reference for “He et al., 2019” was erroneously written as “He, X., Li, J. J., Chen, Y., Yang, J. Q., and Chen, X. Y. (2019). Genome-wide analysis of the WRKY gene family and its response to abiotic stress in buckwheat (Fagopyrum tataricum). *Open Life Sci.* 14, 80–96. doi: 10.1515/biol-2019-0011”. It should be “He, X., Li, J. J., Chen, Y., Yang, J. Q., and Chen, X. Y. (2019). Genome-wide analysis of the WRKY gene family and its response to abiotic stress in buckwheat (*Fagopyrum tataricum*). *Open Life Sci*. 14, 80–96. doi: 10.1515/biol-2019-0010”.
- The reference for “Hou et al., 2016” was erroneously written as “Hou, S., Sun, Z., Linghu, B., Xu, D., Wu, B., Zhang, B., et al. (2016). Genetic diversity of buckwheat cultivars (Fagopyrum tartaricum, Gaertn.) assessed with SSR markers developed from genome survey sequences. *Plant Mol. Biol. Rep.* 34, 233–241. doi: 10.1007/s11105-015-0935-1”. It should be “Hou, S., Sun, Z., Linghu, B., Xu, D., Wu, B., Zhang, B., et al. (2016). Genetic diversity of buckwheat cultivars (*Fagopyrum tartaricum*, Gaertn.) assessed with SSR markers developed from genome survey sequences. *Plant Mol. Biol. Rep*. 34, 233–241. doi: 10.1007/s11105-015-0907-5”.
- The reference for “Imamura et al., 2019” was erroneously written as “Imamura, T., Isozumi, N., Higashimura, Y., Miyazato, A., Mizukoshi, H., Ohki, S.,et al. (2019). Isolation of amaranthin synthetase from Chenopodium quinoa and construction of an amaranthin production system using suspension-cultured tobacco BY-2 cells. Plant Biotechnol. J. 17, 969–981. doi: 10.1111/pbi.13017”. It should be “Imamura, T., Isozumi, N., Higashimura, Y., Miyazato, A., Mizukoshi, H., Ohki, S., et al. (2019). Isolation of amaranthin synthetase from Chenopodium quinoa and construction of an amaranthin production system using suspension-cultured tobacco BY-2 cells. Plant Biotechnol. J. 17, 969–981. doi: 10.1111/pbi.13032”.
- The reference for “Islam et al., 2016” was erroneously written as “Islam, M. S., Tahjib-Ul-Arif, M., Islam, M. A., Hossain, M. A., Siddiqui, M. N., and Sayed, M. A. (2016). Dietary effects of buckwheat (Fagopyrum esculentum) and black cumin (Nigella sativa) seed on growth performance, serum lipid profile, and intestinal microflora of broiler chicks. South Afr. J. Anim. Sci 46, 103–111. doi: 10.4314/sajas.v46i1.12”. It should be “Islam, M. S., Tahjib-Ul-Arif, M., Islam, M. A., Hossain, M. A., Siddiqui, M. N., and Sayed, M. A. (2016). Dietary effects of buckwheat (*Fagopyrum esculentum*) and black cumin (*Nigella sativa*) seed on growth performance, serum lipid profile, and intestinal microflora of broiler chicks. *South Afr. J. Anim. Sci.* 46, 103–111. doi: 10520/EJC187295”.
- The reference for “Kolano et al., 2013” was erroneously written as “Kolano, B., Bednara, E., and Weiss-Schneeweiss, H. (2013). Isolation and characterization of reverse transcriptase fragments of LTR retrotransposons from the genome of Chenopodium quinoa (Amaranthaceae). Plant Cell Rep. 32, 1575–1588. doi: 10.1007/s00299-013-1478-1”. It should be “Kolano, B., Bednara, E., and Weiss-Schneeweiss, H. (2013). Isolation and characterization of reverse transcriptase fragments of LTR retrotransposons from the genome of *Chenopodium quinoa* (Amaranthaceae). Plant Cell Rep. 32, 1575–1588. doi: 10.1007/s00299-013-1468-4”.
- The reference for “Leiber, 2016” was erroneously written as “Leiber, F. (2016). “Buckwheat in the nutrition of livestock and poultry,” in Molecular Breeding and Nutritional Aspects of Buckwheat (Amsterdam: Academic Press), pp.229 pp.238”. It should be “Leiber, F. (2016). “Buckwheat in the nutrition of livestock and poultry,” in Molecular Breeding and Nutritional Aspects of Buckwheat (Amsterdam: Academic Press), pp.229–pp.238. doi: 10.1016/B978-0-12-803692-1.00018-3”.
- The reference for “Lemos et al., 2012” was erroneously written as “Lemos, A. D. R., Capriles, V. D., Pinto e Silva, M. E. M., and Arêas, J. A. G. (2012). Effect of incorporation of amaranth on the physical properties and nutritional value of cheese bread. *Food Sci Technol.* 32, 427–431. doi: 10.1590/S0101-20612012005000057”. It should be “Lemos, A. D. R., Capriles, V. D., Pinto e Silva, M. E. M., and Arêas, J. A. G. (2012). Effect of incorporation of amaranth on the physical properties and nutritional value of cheese bread. *Food Sci Technol.* 32, 427–431. doi: 10.1590/S0101-20612012005000079”.
- The reference for “Mizuno and Yasui, 2019” was erroneously written as “Mizuno, N., and Yasui, Y. (2019). Gene flow signature in the S-allele region of cultivated buckwheat. *BMC Plant Biol.* 19, 125. doi: 10.1186/s12870-019-1739-4”. It should be “Mizuno, N., and Yasui, Y. (2019). Gene flow signature in the S-allele region of cultivated buckwheat. *BMC Plant Biol.* 19, 125. doi: 10.1186/s12870-019-1730-1”.
- The reference for “Palmeros-Suarez etal., 2015” was erroneously written as “Palmeros-Suarez, P. A., Massange-Sanchez, J. A., Martınez-Gallardo, N. A., Montero-Vargas, J. M., Gomez-Leyva, J. F., and Delano-Frier, J. P. (2015). The overexpression of an Amaranthus hypochondriacus NF-YC gene modifies growth and confers water deficit stress resistance in Arabidopsis. *Plant Sci.* 240, 25–40. doi: 10.1016/j.plantsci.2015.08.007”. It should be “Palmeros-Suarez, P. A., Massange-Sanchez, J. A., Martınez-Gallardo, N. A., Montero-Vargas, J. M., Gomez-Leyva, J. F., and Delano-Frier, J. P. (2015). The overexpression of an *Amaranthus hypochondriacus* NF-YC gene modifies growth and confers water deficit stress resistance in Arabidopsis. *Plant Sci.* 240, 25–40. doi: 10.1016/j.plantsci.2015.08.010”.
- The reference for “Qin et al., 2013” was erroneously written as “Qin, P., Li, W., Yang, Y., and Guixing, R. (2013). Changes in phytochemical compositions, antioxidant and a-glucosidase inhibitory activities during the processing of tartary buckwheat tea. Food Res. Int. 50, 562–567. doi: 10.1016/j.foodres.2012.10.037”. It should be “Qin, P., Li, W., Yang, Y., and Guixing, R. (2013). Changes in phytochemical compositions, antioxidant and a-glucosidase inhibitory activities during the processing of tartary buckwheat tea. *Food Res. Int.* 50, 562–567. doi: 10.1016/j.foodres.2011.03.028”.
- The reference for “Rahman et al., 2024” was erroneously written as “Rahman, H., Vikram, P., Hu, Y., Asthana, S., Tanaji, A., Suryanarayanan, P., et al. (2024). Mining genomic regions associated with agronomic and biochemical traits in quinoa through GWAS. *Sci. Rep.* 14, 9205. doi: 10.1038/s41598-024-58307-9”. It should be “Rahman, H., Vikram, P., Hu, Y., Asthana, S., Tanaji, A., Suryanarayanan, P., et al. (2024). Mining genomic regions associated with agronomic and biochemical traits in quinoa through GWAS. *Sci. Rep.* 14, 9205. doi: 10.1038/s41598-024-59565-8”.
- The reference for “Raney et al., 2014” was erroneously written as “Raney, J., Reynolds, D., Elzinga, D., Page, J., Udall, J. A., Jellen, E., et al. (2014). Transcriptome analysis of drought induced stress in *Chenopodium quinoa*. *Am. J. Plant Sci.* 5, 338–357. doi: 10.4236/ajps.2014.53038”. It should be “Raney, J., Reynolds, D., Elzinga, D., Page, J., Udall, J. A., Jellen, E., et al. (2014). Transcriptome analysis of drought induced stress in *Chenopodium quinoa*. *Am. J. Plant Sci.* 5, 338–357. doi: 10.4236/ajps.2014.53047”.
- The reference for “Ruiz Carrasco et al., 2011” was erroneously written as “Ruiz-Carrasco, K., Antognoni, F., Coulibaly, A. K., Lizardi, S., Covarrubias, A., Martınez, E. A., et al. (2011). Variation in salinity tolerance of four lowland genotypes of quinoa (Chenopodium quinoa Willd.) as assessed by growth, physiological traits, and sodium transporter gene expression. *Plant Physiol. Biochem.* 49, 1333–1341. doi: 10.1016/j.plaphy.2011.08.003”. It should be “Ruiz-Carrasco, K., Antognoni, F., Coulibaly, A. K., Lizardi, S., Covarrubias, A., Martınez, E. A., et al. (2011). Variation in salinity tolerance of four lowland genotypes of quinoa (*Chenopodium quinoa* Willd.) as assessed by growth, physiological traits, and sodium transporter gene expression. *Plant Physiol. Biochem.* 49, 1333–1341. doi: 10.1016/j.plaphy.2011.08.005”.
- The reference for “Saad-Allah and Youssef, 2018” was erroneously written as “Saad-Allah, K. M., and Youssef, M. S. (2018). Phytochemical and genetic characterization of five quinoa (Chenopodium quinoa Willd.) genotypes introduced to Egypt. Physiol. Mol. Biol. Plants 24, 617–629. doi: 10.1007/s12298-018-0527”. It should be “Saad-Allah, K. M., and Youssef, M. S. (2018). Phytochemical and genetic characterization of five quinoa (*Chenopodium quinoa* Willd.) genotypes introduced to Egypt. Physiol. Mol. Biol. Plants 24, 617-629. doi: 10.1007/s12298-018-0541-4”.
- The reference for “Schoenlechner et al., 2010” was erroneously written as “Schoenlechner, R., Wendner, M., Siebenhandl-Ehn, S., and Berghofer, E. (2010). Pseudocereals as alternative sources for high folate content in staple foods. *J. Cereal Sci.* 52, 475–479. doi: 10.1016/j.jcs.2010.08.007”. It should be “Schoenlechner, R., Wendner, M., Siebenhandl-Ehn, S., and Berghofer, E. (2010). Pseudocereals as alternative sources for high folate content in staple foods. *J. Cereal Sci.* 52, 475–479. doi: 10.1016/j.jcs.2010.08.001”.
- The reference for “Takeshima et al., 2019” was erroneously written as “Takeshima, R., Nishio, T., Komatsu, S., Kurauchi, N., and Matsui, K. (2019). Identification of a gene encoding polygalacturonase expressed specifically in short styles in distylous common buckwheat (Fagopyrum esculentum). *Heredity* 123, 492–502. doi: 10.1038/s41437-019-0239-3”. It should be “Takeshima, R., Nishio, T., Komatsu, S., Kurauchi, N., and Matsui, K. (2019). Identification of a gene encoding polygalacturonase expressed specifically in short styles in distylous common buckwheat (*Fagopyrum esculentum*). *Heredity* 123, 492–502. doi: 10.1038/s41437-019-0227-x”.
- The reference for “Wang et al., 2014” was erroneously written as “Wang, X., Feng, B., Xu, Z., Sestili, F., Zhao, G., Xiang, C., et al. (2014). Identification and characterization of granule bound starch synthase I (GBSSI) gene of tartary buckwheat (Fagopyrum tataricum Gaertn.). *Gene* 534, 229–235.doi: 10.1016/j.gene.2013.11.076”. It should be “Wang, X., Feng, B., Xu, Z., Sestili, F., Zhao, G., Xiang, C., et al. (2014). Identification and characterization of granule bound starch synthase I (GBSSI) gene of tartary buckwheat (*Fagopyrum tataricum* Gaertn.). *Gene* 534, 229–235. doi: 10.1016/j.gene.2013.10.053”.
- The reference for “Wu et al., 2019” was erroneously written as “Wu, Q., Zhao, G., Bai, X., Wei, Z., Xiang, D., Wan, Y., et al. (2019). Characterization of the transcriptional profiles in common buckwheat (Fagopyrum esculentum) under PEG-mediated drought stress. *Electronic J. Biotechnol.* 39, 42–51. doi: 10.1016/j.ejbt.2019.04.006”. It should be “Wu, Q., Zhao, G., Bai, X., Wei, Z., Xiang, D., Wan, Y., et al. (2019). Characterization of the transcriptional profiles in common buckwheat (*Fagopyrum esculentum*) under PEG-mediated drought stress. *Electronic J. Biotechnol.* 39, 42–51. doi: 10.1016/j.ejbt.2019.03.005”.
- The reference for “Xiao-Lin et al., 2022” was erroneously written as “Xiao-Lin, Z., Bao-Qiang, W., and Xiao-Hong, W. (2022). Identification and expression analysis of the CqSnRK2 gene family and a functional study of the CqSnRK2.12 genes in quinoa (Chenopodium quinoa Willd.). *BMC Genomics* 23, 397. doi: 10.1186/s12864-022-08688-5”. It should be “Xiao-Lin, Z., Bao-Qiang, W., and Xiao-Hong, W. (2022). Identification and expression analysis of the CqSnRK2 gene family and a functional study of the CqSnRK2.12 genes in quinoa (*Chenopodium quinoa* Willd.). *BMC Genomics* 23, 397. doi: 10.1186/s12864-022-08626-1”.
- The reference for “Xie et al., 2023” was erroneously written as “Xie, H., Zhang, P., Jiang, C., Wang, Q., Guo, Y., Zhang, X., et al. (2023). Combined transcriptomic and metabolomic analyses of high temperature stress response of quinoa seedlings. *BMC Plant Biol.* 23, 292. doi: 10.1186/s12870-023-04377-1”. It should be “Xie, H., Zhang, P., Jiang, C., Wang, Q., Guo, Y., Zhang, X., et al. (2023). Combined transcriptomic and metabolomic analyses of high temperature stress response of quinoa seedlings. *BMC Plant Biol.* 23, 292. doi: 10.1186/s12870-023-04310-y”.
- The reference for “Xu et al., 2020” was erroneously written as “Xu, J., Zhang, Y., Wang, W., and Li, Y. (2020). Advanced properties of gluten-free cookies, cakes, and crackers: A review. *Trends Food Sci Technol.* 103, 200–213. doi: 10.1016/j.tifs.2020.07.004”. It should be “Xu, J., Zhang, Y., Wang, W., and Li, Y. (2020). Advanced properties of gluten-free cookies, cakes, and crackers: A review. *Trends Food Sci Technol.* 103, 200–213. doi: 10.1016/j.tifs.2020.07.017”.
- The reference for “Yao et al., 2017” was erroneously written as “Yao, H. P., Li, C. L., Zhao, H. X., Zhao, J., Chen, H., Bu, T., et al. (2017). Deep sequencing of the transcriptome reveals distinct flavonoid metabolism features of black tartary buckwheat (Fagopyrum tataricum Gaertn.). *Prog. Biophysics Mol. Biol.* 124, 49–60. doi: 10.1016/j.pbiomolbio.2016.11.005”. It should be “Yao, H. P., Li, C. L., Zhao, H. X., Zhao, J., Chen, H., Bu, T., et al. (2017). Deep sequencing of the transcriptome reveals distinct flavonoid metabolism features of black tartary buckwheat (*Fagopyrum tataricum* Gaertn.). *Prog. Biophysics Mol. Biol.* 124, 49–60. doi: 10.1016/j.pbiomolbio.2016.11.003”.
- The reference for “Yeo et al., 2015” was erroneously written as “Yeo, M. T., Carella, P., Fletcher, J., Champigny, M. J., Weretilnyk, E. A., and Cameron, R. K. (2015). Development of a Pseudomonas syringae–Eutrema salsugineum pathosystem to investigate disease resistance in a stress tolerant extremophile model plant. *Plant Pathol.* 64, 297–306. doi: 10.1111/ppa.12235”. It should be “Yeo, M. T., Carella, P., Fletcher, J., Champigny, M. J., Weretilnyk, E. A., and Cameron, R. K. (2015). Development of a *Pseudomonas syringae*–*Eutrema salsugineum* pathosystem to investigate disease resistance in a stress tolerant extremophile model plant. *Plant Pathol.* 64, 297–306. doi: 10.1111/ppa.12271”.
- The reference for “Yokosho et al., 2014” was erroneously written as “Yokosho, K., Yamaji, N., and Ma, J. F. (2014). Global transcriptome analysis of Al-induced genes in an Al-accumulating species, common buckwheat (Fagopyrum esculentum Moench). Plant Cell Physiol. 55, 2077–2091. doi: 10.1093/pcp/pcu157”. It should be “Yokosho, K., Yamaji, N., and Ma, J. F. (2014). Global transcriptome analysis of Al-induced genes in an Al-accumulating species, common buckwheat (*Fagopyrum esculentum* Moench). Plant Cell Physiol. 55, 2077–2091. doi: 10.1093/pcp/pcu135”.
- The reference for “Zamaratskaia et al., 2023” was erroneously written as “Zamaratskaia, G., Egelandsdal, B., and Karlsson, A. H. (2023). Nutritional properties and potential applications of buckwheat in plant-based foods. *Trends Food Sci Technol.* 139, 124–132. doi: 10.1016/j.tifs.2023.01.008”. It should be “Sonawane, S., Shams, R., Dash, K. K., Patil, V., Pandey, V. K., & Dar, A. H. (2024). Nutritional profile, bioactive properties and potential health benefits of buckwheat: A review. *EFood* 5 (4), e171. doi: 10.1002/efd2.171”.
- The reference for “Zhang et al., 2021” was erroneously written as “Zhang, K., He, M., Fan, Y., Zhao, H., Gao, B., Yang, K., et al. (2021). Resequencing of global Tartary buckwheat accessions reveals multiple domestication events and key loci associated with agronomic traits. *Genome Biol.* 22, 1 17. doi: 10.1186/s13059-020-02237-5”. It should be “Zhang, K., He, M., Fan, Y., Zhao, H., Gao, B., Yang, K., et al. (2021). Resequencing of global Tartary buckwheat accessions reveals multiple domestication events and key loci associated with agronomic traits. *Genome Biol.* 22, 1 17. doi: 10.1186/s13059-020-02217-7”.
- The reference for “Zhang et al., 2017” was erroneously written as “Zhang, L., Li, X., Ma, B., Gao, Q., Du, H., Han, Y., et al. (2017). The tartary buckwheat genome provides insights into rutin biosynthesis and abiotic stress tolerance. *Mol. Plant* 10, 1224–1237. doi: 10.1016/j.molp.2017.08.008”. It should be “Zhang, L., Li, X., Ma, B., Gao, Q., Du, H., Han, Y., et al. (2017). The tartary buckwheat genome provides insights into rutin biosynthesis and abiotic stress tolerance. *Mol. Plant* 10, 1224–1237. doi: 10.1016/j.molp.2017.08.013”.
- The reference for “Zhao et al., 2023” was erroneously written as “Zhao, H., He, Y., Zhang, K., Li, S., Chen, Y., He, M., et al. (2023). Rewiring of the seed metabolome during Tartary buckwheat domestication. *Plant Biotechnol.* J. 21, 150–164. doi: 10.1111/pbi.13988”. It should be “Zhao, H., He, Y., Zhang, K., Li, S., Chen, Y., He, M., et al. (2023). Rewiring of the seed metabolome during Tartary buckwheat domestication. *Plant Biotechnol.* J. 21, 150–164. doi: 10.1111/pbi.13932”.
- The reference for “Zhu et al., 2015” was erroneously written as “Zhu, H., Wang, H., Zhu, Y., Zou, J., Zhao, F. J., and Huang, C. F. (2015). Genome-wide transcriptomic and phylogenetic analyses reveal distinct aluminum-tolerance mechanisms in the aluminum accumulating species buckwheat (Fagopyrum tataricum). *BMC Plant Biol.* 15, 16. doi: 10.1186/s12870-015-0417-9”. It should be “Zhu, H., Wang, H., Zhu, Y., Zou, J., Zhao, F. J., and Huang, C. F. (2015). Genome-wide transcriptomic and phylogenetic analyses reveal distinct aluminum-tolerance mechanisms in the aluminum accumulating species buckwheat (*Fagopyrum tataricum*). *BMC Plant Biol.* 15, 16. doi: 10.1186/s12870-014-0395-z”.

The reference “Woomer, J. S., and Adedeji, A. A. (2020). Current applications of gluten-free grains –a review. Crit. Rev. Food Sci Nutr. 61, 14–24. doi: 10.1080/10408398.2020.1713724” was duplicated in the reference list. The duplication has been removed.

A correction has been made to the section **Pseudocereals in food security**, paragraph 1. The year in “Alencar and de Carvalho Oliveira, 2023” should be “2019” instead. The text has been updated to read as follows:

“After processing, pseudocereals are used in baked goods, fermented drinks, and extruded snacks (Alencar and de Carvalho Oliveira, 2019; Graziano et al., 2022; Martínez-Villaluenga et al., 2020)”.

A correction has been made to section **Pseudocereals as alternative crops**, paragraph 1. Instead of: “The figure depicts that pseudocereals contribute only 14%, indicating their significant role as alternative crops in different food groups”, the line should read: “The figure depicts that pseudocereals contribute only 14 species, indicating their significant role as alternative crops in different food groups”.

**Figure 2:**
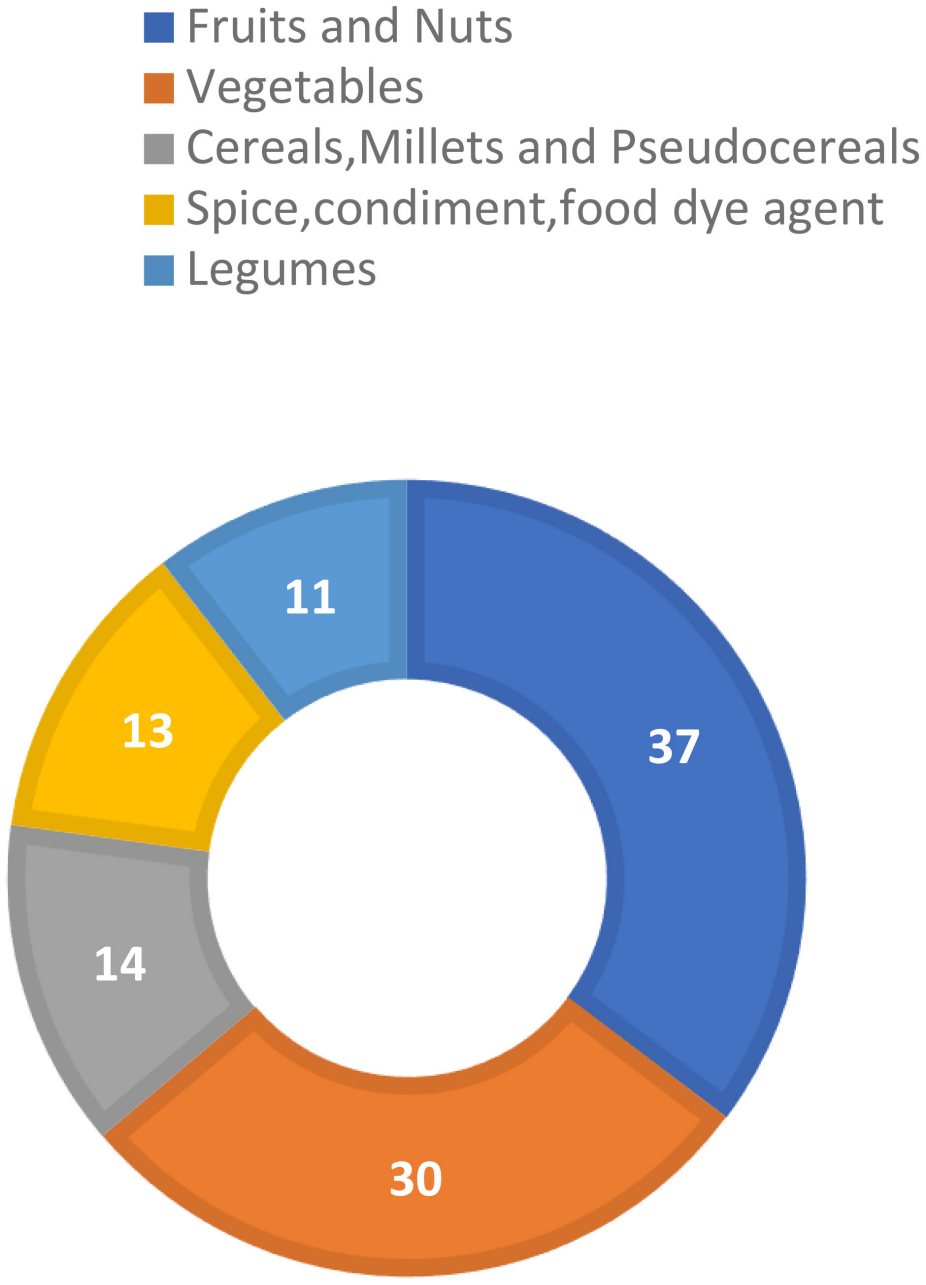
Pseudocereals’ contribution in alternative food groups. (Modified from Pradhan et al., 2021).

There was a mistake in **Figure 2** and its caption as published. The percentage symbol “%” should be removed from the pie chart numbers and the caption “Pseudocereals’ contribution in alternative food groups Source: Indian Food Composition Tables, NIN (Longvah, 2017)” should be changed with: “Pseudocereals’ contribution in alternative food groups. (Modified from Pradhan et al., 2021)”. The reference details for Pradhan et al., 2021 are: “Pradhan, A., Rane, J., & Pathak, H. (2021). Alternative crops for augmenting farmers’ income in abiotic stress regions. *Technical Bulletin*, (29), 26.”

The corrected **Figure 2** and its caption appear below.

The original version of this article has been updated.

